# Intracellular Cytokine Staining and Flow Cytometry: Considerations for Application in Clinical Trials of Novel Tuberculosis Vaccines

**DOI:** 10.1371/journal.pone.0138042

**Published:** 2015-09-14

**Authors:** Steven G. Smith, Kaatje Smits, Simone A. Joosten, Krista E. van Meijgaarden, Iman Satti, Helen A. Fletcher, Nadia Caccamo, Francesco Dieli, Francoise Mascart, Helen McShane, Hazel M. Dockrell, Tom H. M. Ottenhoff, TBVI TB Biomarker Working Group

**Affiliations:** 1 Department of Immunology and Infection, Faculty of Infectious and Tropical Diseases, London School of Hygiene and Tropical Medicine, Keppel Street, London, United Kingdom; 2 Laboratory of Vaccinology and Mucosal Immunity, Université Libre de Bruxelles, Brussels, Belgium; 3 Department of Infectious Diseases, Leiden University Medical Center, Leiden, The Netherlands; 4 The Jenner Institute, Old Road Campus Research Building, Roosevelt Drive, Oxford, United Kingdom; 5 Biomedical Research Centre, Università di Palermo, Palermo, Italy, Dipartimento di Biopatologia e Biotecnologie Mediche e Forensi, Università di Palermo, Palermo, Italy; 6 Immunobiology Clinic, Hôpital Erasme, Université Libre de Bruxelles (U.L.B.), Brussels, Belgium; University of Cape Town, SOUTH AFRICA

## Abstract

Intracellular cytokine staining combined with flow cytometry is one of a number of assays designed to assess T-cell immune responses. It has the specific advantage of enabling the simultaneous assessment of multiple phenotypic, differentiation and functional parameters pertaining to responding T-cells, most notably, the expression of multiple effector cytokines. These attributes make the technique particularly suitable for the assessment of T-cell immune responses induced by novel tuberculosis vaccines in clinical trials. However, depending upon the particular nature of a given vaccine and trial setting, there are approaches that may be taken at different stages of the assay that are more suitable than other alternatives. In this paper, the Tuberculosis Vaccine Initiative (TBVI) TB Biomarker Working group reports on efforts to assess the conditions that will determine when particular assay approaches should be employed. We have found that choices relating to the use of fresh whole blood or peripheral blood mononuclear cells (PBMC) and frozen PBMC; use of serum-containing or serum-free medium; length of stimulation period and use of co-stimulatory antibodies can all affect the sensitivity of intracellular cytokine assays. In the case of sample material, frozen PBMC, despite some loss of sensitivity, may be more advantageous for batch analysis. We also recommend that for multi-site studies, common antibody panels, gating strategies and analysis approaches should be employed for better comparability.

## Introduction

In clinical vaccine studies and trials, monitoring of vaccine-induced immunity is essential. As well as providing a measure of vaccine take in individuals, immunological biomarkers that change with vaccine interventions may be candidate correlates of protection themselves, or may help focus the search for reliable correlates on the relevant immune mechanisms.

A number of assays exist that allow the measurement of immunological biomarkers in material derived from venous blood, the most accessible tissue for immunological evaluation in clinical trials, and many of these assays have been discussed elsewhere [1–5]. Intracellular cytokine staining (ICS) of stimulated peripheral blood mononuclear cells (PBMC) followed by flow cytometric analysis is a well-established method for detecting immunological biomarkers in the form of expressed cytokines. Unlike alternative approaches that also detect cytokine expression such as enzyme-linked immunospot (ELISpot) or ELISA assays, ICS enables the simultaneous detection of the specific subset of responder cells (e.g. CD4 or CD8 positive T-cells); of associated markers of differentiation (e.g. markers of memory phenotype or activation state) and function (e.g. cytokine production, cytotoxicity-associated markers etc.); multiple cytokines/chemokines simultaneously and of markers of proliferation. Modern multi-parameter instruments increasingly allow for the measurement of simultaneous expression of numerous markers, such as the presence of multiple cytokines or effector molecules that characterise the so-called polyfunctional T-cell phenotypes [6–8]. Advanced and flexible functionality such as this is essential in modern vaccine development where, for a disease such as tuberculosis, different vaccine candidates target different cell populations and cytokine responses (Table 1).

**Table 1:** Expected/targeted immune responses of novel TB vaccine candidates.

Vaccine candidate	Vaccine format	Immune response targeted/expected
*M*. *indicus pranii*[9]	Whole-cell *M*. *indicus pranii*	CD4^+^ and CD8^+^ T-cell responses; antibody response; innate response; non-conventional T-cells
*M*. *vaccae*[10]	Whole-cell *M*. *vaccae*	CD4^+^ and CD8^+^ T-cell responses; antibody response; innate response; non-conventional T-cells
MVA85A[11]	Recombinant viral vector	CD4^+^ high and CD8^+^ T-cell low responses
M72 + AS01[12,13]	Protein/adjuvant	CD4^+^ high and CD8^+^ T-cell responses
VPM1002[14]	Live recombinant recombinant BCG	CD4^+^ high and CD8^+^ T-cell responses; antibody response; innate response; non-conventional T-cells
Crucell Ad35[15]	Recombinant viral vector	CD4^+^ low and CD8^+^ T-cell high responses
Hybrid 1 + IC31[16]	Protein/adjuvant	CD4^+^ high and CD8^+^ T-cell responses
Hybrid 4 + IC31[17,18]	Protein/adjuvant	CD4^+^ high and CD8^+^ T-cell responses
Hybrid 56 + IC31[19,20]	Protein/adjuvant	CD4^+^ high and CD8^+^ T-cell responses
RUTI[21]	Fragmented MTB	CD4^+^ and CD8^+^ T-cell responses; antibody response; innate response
Ad5Ag85A[22]	Recombinant viral vector	CD4^+^ low and CD8^+^ T-cell high responses
ChAdOx1 85A + MVA85A (www.aeras.org/candidates)	Recombinant viral vector	CD4^+^ and CD8^+^ T-cell responses
Combination Crucell Ad35 + MVA85A (www.aeras.org/candidates)	Recombinant viral vector	CD4^+^ high and CD8^+^ T-cell responses
DAR-901 (www.aeras.org/candidates)	Whole-cell *M*. *obuense*	CD4^+^ and CD8^+^ T-cell responses; antibody response; innate response; non-conventional T-cells
ID93 + GLA-SE (www.aeras.org/candidates)	Protein/adjuvant	CD4^+^ high and CD8^+^ T-cell responses
MTBVAC[23]	Live genetically attenuated MTB	CD4^+^ and CD8^+^ T-cell responses; antibody response; innate response; non-conventional T-cells

Unlike ELISpot and ELISA assays that comprise a series of well-defined steps and are easily packaged into a kit format, ICS assays have arisen much more organically in different laboratories where different steps have been optimised to work with the particular tissues, stimulants, cell phenotypes and cytokines of interest for each group and setting as well as the different instruments and laser configurations available. Therefore, when ICS is to be used to measure immune responses as part of a clinical trial of a novel vaccine, the ICS assay protocol must be optimised at each step for the specific analysis that is intended; to suit the clinical material available; and to fit into the working environment of the trial. With the number of possible analytes increasing using state-of-the-art methodologies (15+ colour flow cytometers), the cell populations to be analysed become smaller; for ICS, percentages of 0.1% positive events or less are now commonly reported, urging the need for highly reproducible and standardised results.

This manuscript reports on the lessons drawn from the activities of a flow cytometry working group comprised of participants in the human TB biomarkers work package of the European Commission FP7-funded NEWTBVAC consortium project, which is part of TBVI (www.tbvi.eu). As well as conducting research into potential biomarkers of TB risk, protection and disease, the group compared the specifics of the ICS/flow cytometry assays that are increasingly used to measure such biomarkers. Each stage of the experimental protocol is discussed and where different approaches are available, recommendations are made as to best practice, based on data arising out of this collaborative project.

## Materials and Methods

### Ethical approval

The use of human blood samples in experiments described in this paper was approved either by the Ethics Committee of the London School of Hygiene and Tropical Medicine (UK) (ref. 5520) or by the Ethics Committee Universite Libre de Bruxelles–Hospital Erasme (Belgium) (ref. P2011-113). Informed written consent was obtained from all participants.

### Antigen stimulation

PBMC were isolated and stimulated as previously described [24] or in some experiments, venous blood was diluted 1:1 with warm Iscove’s Modified Dulbecco’s Medium (Lonza, Belgium) and stimulated directly. Where frozen PBMC were thawed and used, these were previously cryopreserved at -80°C in freezing medium (RPMI 1640 (Invitrogen) + 20% foetal calf serum (Sigma) + 10% dimethylsulphoxide (Sigma)). PBMC (fresh or thawed) or diluted whole blood was stimulated for between 18 hours and five days in different experiments with heparin-binding haemaggluttinin (HBHA) (10μg/ml; purified as previously described [25]), recombinant ESAT-6 protein (Lionex, Braunschweig, Germany; 10μg/ml), purified protein derivative (PPD; Statens Serum Institute, Copenhagen, Denmark; 4μg/ml or 10μg/ml as indicated) or *Staphylococcus* enterotoxin B (SEB; Sigma–Aldrich, St. Louis, MO; 0.5μg/ml). In some experiments, anti-CD28 and anti-CD49d co-stimulatory antibodies (BD Biosciences, Oxford, UK) were added at 2 μg/ml

### Intracellular cytokine staining and polychromatic flow cytometry

During the last 4 or 16 hours of culture depending upon the experiment, Brefeldin A (3 μg/ml) and Golgistop (1/2000 final dilution; both from BD Biosciences, Mountain View, CA) were added to block cytokine secretion. For diluted whole blood cultures, red cells were lysed with 5 volumes of 1x PharmLyse solution (BD Biosciences, Oxford, UK) for 10 minutes at room temperature. Labeling of dead cells, fixation and permeabilization were performed as previously described [24]. Depending upon the experiment, cells were surface stained with anti-CD4-APC-H7 (BD Biosciences), anti-CD19-efluor450 and anti-CD14-efluor450 (eBiosciences) for 30 minutes at 4°C, or, following permeabilisation, with anti-CD3-Horizon-V500, anti-IL-2-FITC, anti-TNFα-PE-Cy7 (BD Biosciences), anti-IL-17-efluor660, and anti-IFNγ-PerCP-Cy5.5 (Biolegend) for 30 minutes at room temperature. Cells were finally resuspended in 250 μL 1% paraformaldehyde (Sigma, UK) and filtered prior to acquisition on a FACS Canto II flow cytometer or an LSRII flow cytometer (BD Biosciences). Compensation was performed using tubes of CompBeads (BD Biosciences) individually stained with each fluorophor and compensation matrices were calculated with FACSdiva. Data were analyzed using FlowJo software version 9 (Treestar, Ashland, OR). Gating strategy and an example of raw flow cytometry data is shown in S1 Fig.

## Results and Discussion

### Factors to consider when designing ICS/flow cytometry experiments for immune monitoring in vaccine trials

**Fig 1:**
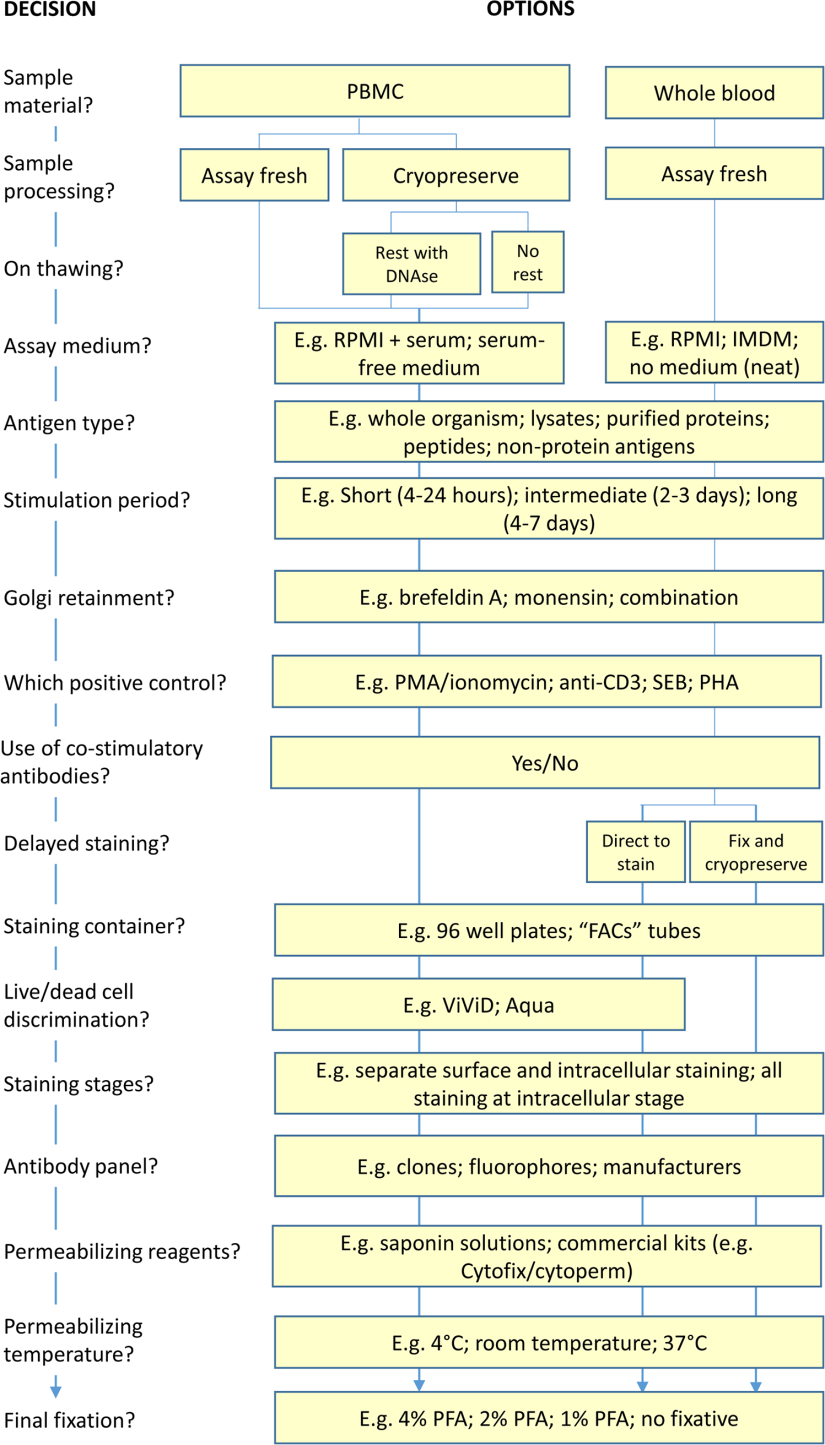
Designing an ICS/flow cytometry experiment. A decision tree presenting, in order that they occur in the protocol, the choices that are required for the formulation of a complete standard operating procedure for the performance of ICS/flow cytometry experiments. Where available, data is provided in this paper to guide some of these decisions.

As many of the variations in ICS assay protocol steps are interdependent, it helps to think of experimental design as a series of decisions that follow on from each other. Such an approach is presented in Fig 1 as a decision tree and we discuss below some of the more pivotal steps contained therein.

#### Sample material

As the most commonly used tissue for immuno-monitoring of responses in clinical vaccine trials is peripheral venous blood, this is the only sample material that will be discussed here, although the applicability of the technique for looking at other immune compartments should be noted e.g. bronchoalveolar lavage [26] or suction blister-derived mononuclear cells [27]. To date, the main target of TB vaccinologists in terms of immunological investigation has been the antigen-specific T-cell population that is relatively easily studied in venous blood. Although the scope of current studies may have expanded to encompass a more comprehensive analysis of the immunological "space", many of the relevant cell populations (B-cell subsets, natural killer cells, monocytes, dendritic cells, neutrophils, innate lymphoid cells, NKT cells, alternative T-cell subsets etc.) are also present in venous blood, hence it remains a valuable source of material for clinical investigation.

There are many advantages to performing assays, including ICS, on freshly drawn venous blood samples. Assays on fresh material may utilize unfractionated whole blood, either undiluted [28] or diluted in tissue culture medium. The advantages of this approach are that it reduces processing and processing related perturbations and allows minimization of the time between blood draw and sample incubations with the relevant stimulants which is important as delayed processing may severely affect the results [1,28]. Moreover, the response measured is the closest possible reflection of the *in vivo* response as all cells (including red blood cells, granulocytes, thrombocytes) but also proteins, lipids, antibodies and other components circulating in the blood are present during the whole blood stimulation assay.

If, rather than whole blood, isolated PBMC are to be used, an additional consideration for trial immunologists is whether to use freshly drawn PBMC for assays or to cryopreserve PBMC aliquots for future batch analysis. One argument for the “fresh” assay approach is the improved response demonstrated when this format is compared to assays performed on cryopreserved equivalent samples which has been observed for lymphocyte proliferation [29]. Although there is no definitive explanation for this observation, possible reasons are proportionally greater losses of activated, effector T-cells and/or of the antigen presenting cells required for the stimulation of responses in recall assays that require antigen processing.

**Fig 2:**
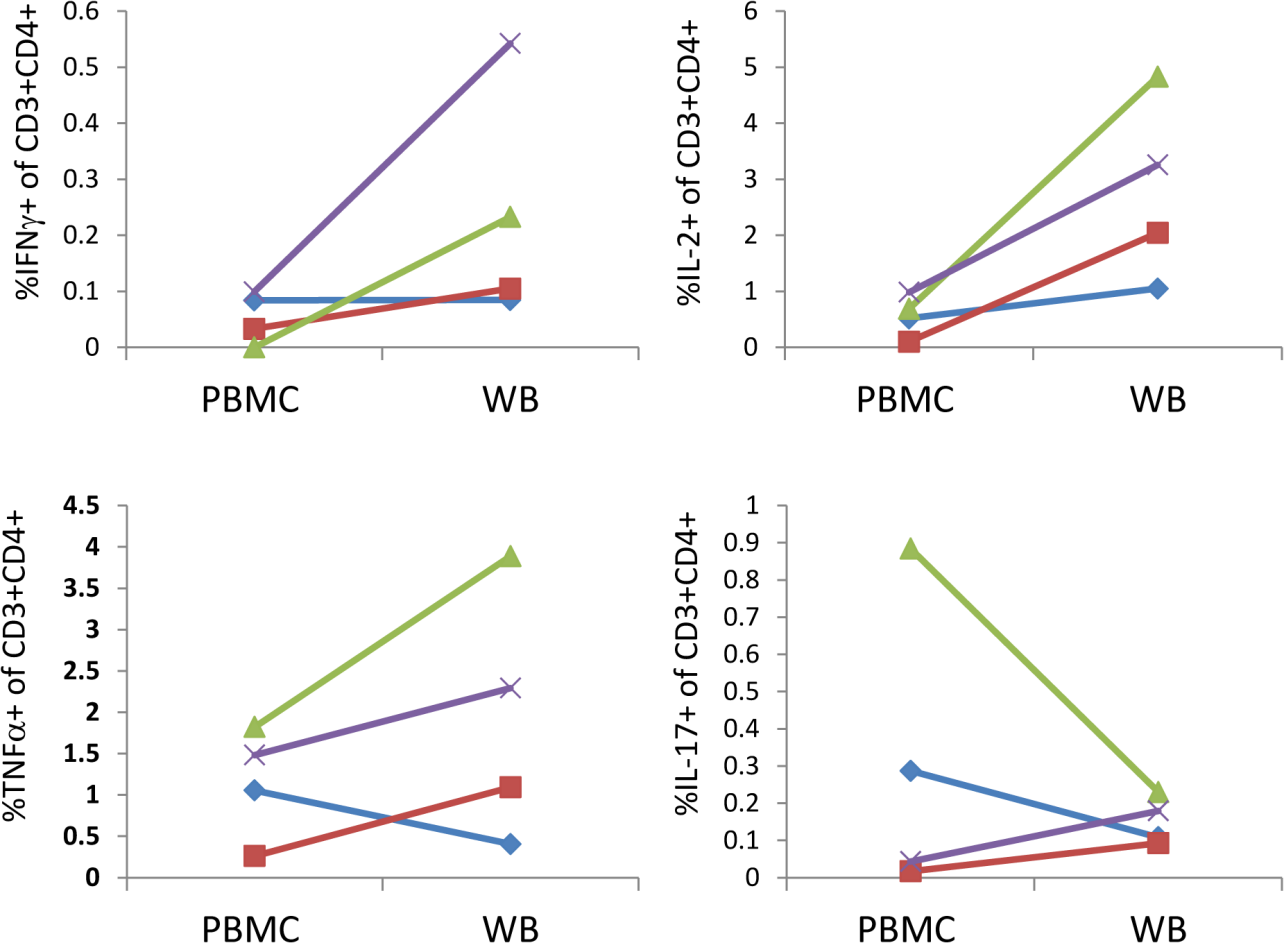
Detection of intracellular cytokines following PBMC or whole blood stimulation. Venous blood samples from 4 healthy donors were stimulated with SEB for 24 hours either as whole blood diluted 1:1 in Iscove’s Modified Dulbecco’s Medium (WB) or following isolation of PBMC by ficoll separation (PBMC). BFA was added for the final 18 hours. Y axes represent the percent of live, single CD3^+^CD4^+^ lymphoid cells that are positive for the indicated cytokine. Lines of different colours and symbols represent individual donors.

**Fig 3:**
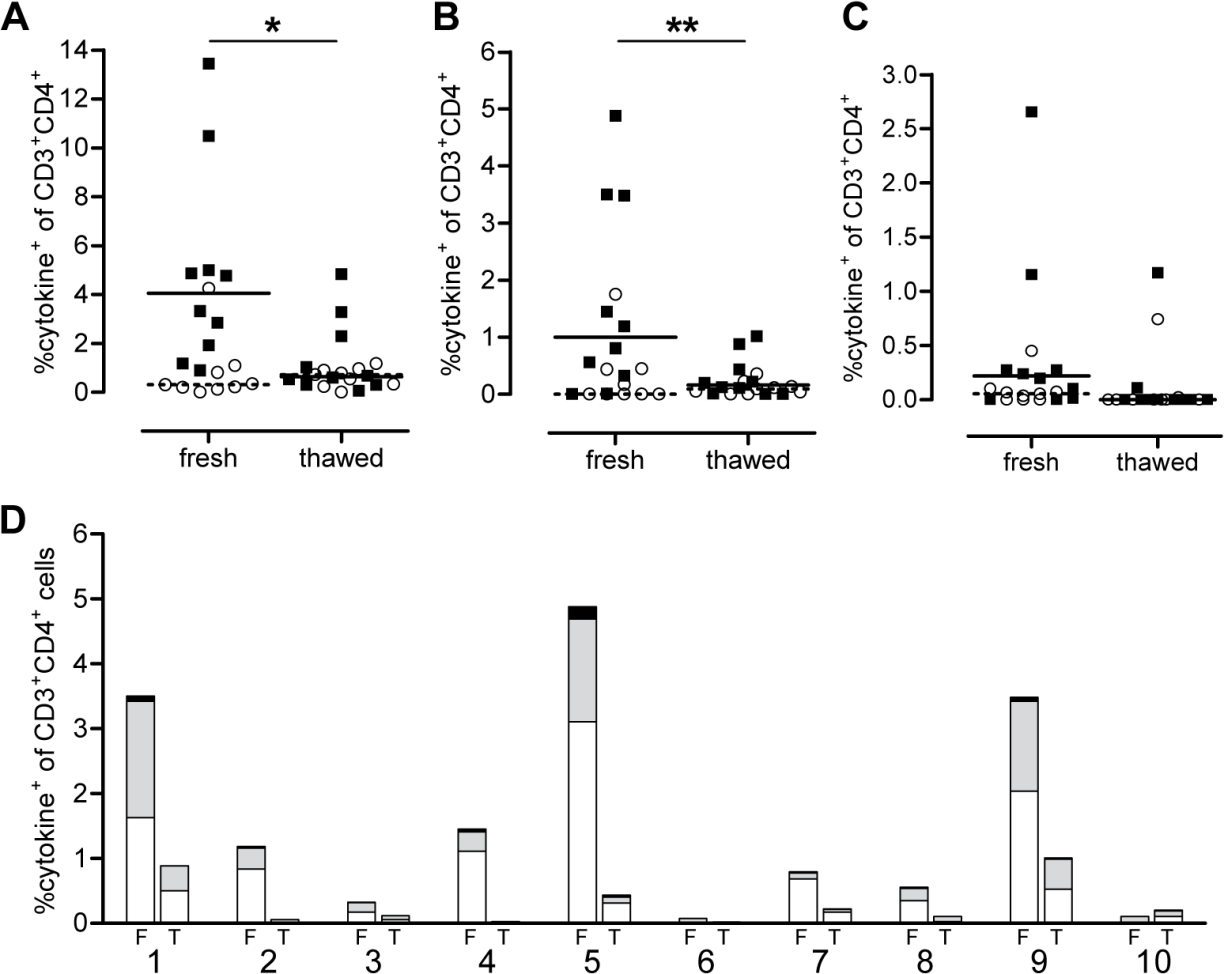
Evaluation of the effect of freezing PBMC for storage on cytokine expression after *in vitro* stimulation for 5 days with *Mtb* antigens. Comparison of total cytokine expression (IFNγ, TNFα and/or IL-2) of CD3^+^CD4^+^ T cells after stimulation of fresh or thawed cells with PPD (A), HBHA (B) and ESAT-6 (C). Open circles and filled squares represent uninfected controls and latent tuberculosis-infected (LTBI) subjects respectively. Horizontal lines represent median frequency of cytokine-expressing cells of controls (dotted) and LTBI subjects (full). (D) For 10 LTBI subjects, the frequency of cytokine-expressing cells is evaluated as single positive (white), double positive (grey) and triple positive (black) cells based on the combination of expressed cytokines (IFNγ, TNFα and IL-2) for fresh (F) and thawed (T) PBMC stimulated with HBHA. Statistical analysis was by Wilcoxon matched pairs signed rank test. * p<0.05, ** p<0.01.

Figs 2 and 3 present data which indicate the general effects on measurable responses of using fresh whole blood or PBMC and of using fresh compared to frozen PBMC. Fig 2 shows a comparison of PBMC and diluted whole blood, both subjected to the same processing time, where, for SEB stimulation, there is a trend for greater frequencies of IFNγ, TNFα and IL-2 secreting cells in whole blood for at least 3 of 4 donors, while the results for IL-17 are more variable. In Fig 3 it can be seen that cytokine expressing cells are generally measurable at higher frequencies when fresh PBMC are stimulated with various antigens as compared to cryopreserved PBMC. The decisions as to whether to use fresh PBMC or whole blood, or whether to use fresh or cryopreserved PBMC will depend on many factors such as the type of stimulant used and the cytokines that are to be detected as well as the logistics of the study and handling of samples. We therefore suggest it is best to carry out pilot studies on both whole blood and PBMC using the specific conditions of a given study, to determine which is optimal for evaluation. It should be noted that here we only show data for SEB stimulation of whole blood versus PBMC and study-specific, antigen stimuli might display different characteristics and should be tested. In addition data for this comparison was only available for 4 donors and so we draw the conclusion that whole blood might give better responses than PBMC with some caution.

Despite the loss in sensitivity seen when using cryopreserved samples, there are a number of compelling reasons why this approach might be favored over the use of fresh samples. When clinical trials are based at facilities with well-equipped laboratories, state-of-the-art flow cytometers, experienced staff, and participants are asked to attend such a facility for the donation of samples, it might be feasible to perform assays on fresh samples. However, in a setting where trials are executed in such a way that laboratory facilities and particularly assay expertise are centralised in a location that might be far removed or even in a different country from the field site, the storage and transportation of frozen PBMC aliquots represents a realistic approach to sample processing if unacceptable losses in sample quality are to be avoided. In such cases, any loss in assay sensitivity following cryopreservation may be compensated by the increase in uniformity with which all sample aliquots will be treated and all assays performed at the centralized facility and the relatively narrow time span in which all samples may be handled. Moreover a major advantage if using banked samples is that all samples from one individual (followed longitudinally) can be tested and evaluated in one experiment, thus ruling out day-to-day variation in assay performance.

One approach that combines the sensitivity of a rapidly performed ‘fresh’ assay with the convenience of cryopreservation and batch processing is to stimulate fresh whole blood and then to fix and freeze samples following Golgi inhibition and cytokine expression but prior to staining and acquisition. This approach (discussed further below) has been used successfully in a number of infant BCG vaccination studies [30–32] as well as in adults (S.A. Joosten, K.E. van Meijgaarden, THM Ottenhoff manuscript submitted for publication).

#### Assay culture medium

**Fig 4:**
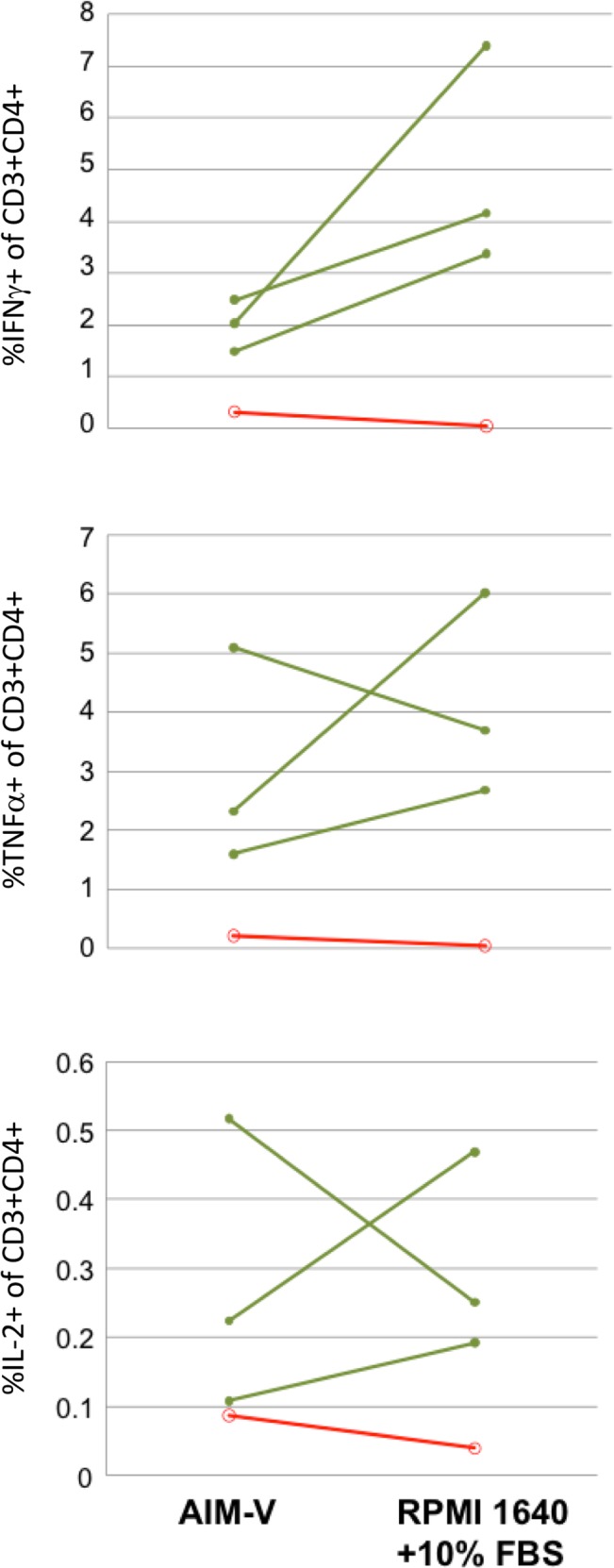
Serum-supplemented or serum-free culture medium for use in ICS assays. PBMC were stimulated with PPD for 5 days in either AIM-V serum-free medium or RPMI 1640 supplemented with 10% foetal bovine serim. BFA and monensin were added for the final 18 hours of the stimulation period. Y-axes represent the percent of live, single CD3^+^CD4^+^ lymphoid cells that are positive for the indicated cytokine. Responses are shown for individuals with latent TB infection (green lines; n = 3) or from healthy, uninfected individuals (red lines; n = 1).

The most commonly used tissue culture media for T-cell assays are RPMI 1640 and Iscove’s modified Dulbecco’s medium (IMDM), usually supplemented with fetal bovine serum (FBS) or human serum (HS). In the interests of uniformity over time and for assays carried out in different places, there is an argument for dispensing with the need for serum supplementation of medium, as serum is more likely to vary between batches in terms of its performance in sensitive T-cell assays. We have compared ICS assays where PBMC were stimulated in either RPMI with 10% FBS or in a serum-free medium alternative (AIM-V). Overall there was no compelling evidence that using RPMI/FBS as compared to AIM-V produced major differences in cytokine responses detected, although there was an increase in the magnitude of IFNγ responses in 3 out of 4 donors when RPMI/FBS was used (Fig 4). The added advantages of using a serum-free medium such as AIM-V (including that it is possible to freeze in aliquots once a large batch has been purchased, for use over long periods) may make this a more attractive option for ICS assays. Moreover, sera-free media should have no background signal derived from circulating analytes, an additional advantage when supernatants from ICS assays are collected for soluble cytokine analysis using ELISA or multiplex immunoassays.

#### Stimulation period

**Fig 5:**
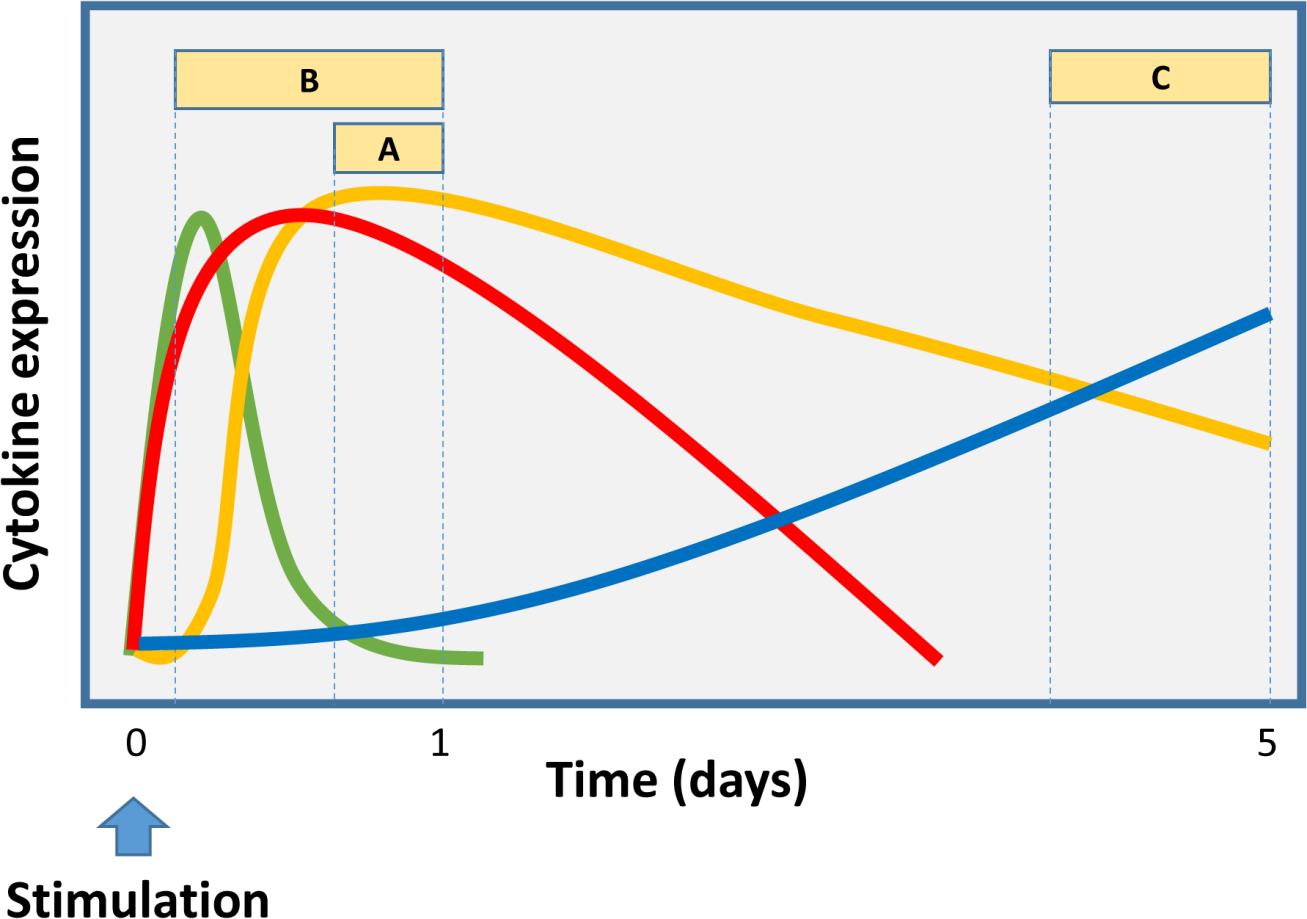
Cytokine expression kinetics and the effect of different Golgi inhibition periods. Three alternative Golgi inhibition periods (A, B and C) with different times of commencement and duration will detect different cytokine secretion profiles when cytokine responses of differing secretion kinetics (green, red, yellow and blue lines) are to be measured. Longer Golgi inhibition periods that start earlier in the stimulation period (i.e. B), are most likely to allow the detection of polyfunctional profiles. A and C are more likely to miss cytokines with early, short expression periods (e.g. green) but later inhibition periods such as C are more likely to detect cytokines with late expression kinetics (e.g. blue).

ICS assays designed to examine the expression of a single cytokine in a given T-cell subset (e.g. CD3^+^ CD4^+^ T-cells), of the type that were commonly performed when 3 and 4 colour flow cytometers were the norm, are relatively easy to optimise in terms of the kinetics of the stimulation and cytokine capture period. For the detection of IFNγ in antigen specific effector T-cells, a 24 hour stimulation period with the final 6 hours in the presence of a Golgi inhibitor such as brefeldin A was common. Since flow cytometers capable of acquiring data from many more detectors simultaneously have been developed, immunologists have turned their attention to cells that are capable of responding to antigen stimulation in a polyfunctional fashion, i.e. with the ability to secrete 3 or more cytokines at once. This however, requires that the stimulation period used as well as the window of secretion inhibition are chosen with the kinetics of expression of all the cytokines of interest considered simultaneously. Fig 5 illustrates a hypothetical experiment that measures the expression of 4 theoretical cytokines by T-cells over time, with each cytokine demonstrating different kinetics of release. Here, this illustration exemplifies the effect of different stimulation periods and secretion inhibition windows on the detection of these cytokines.

**Fig 6:**
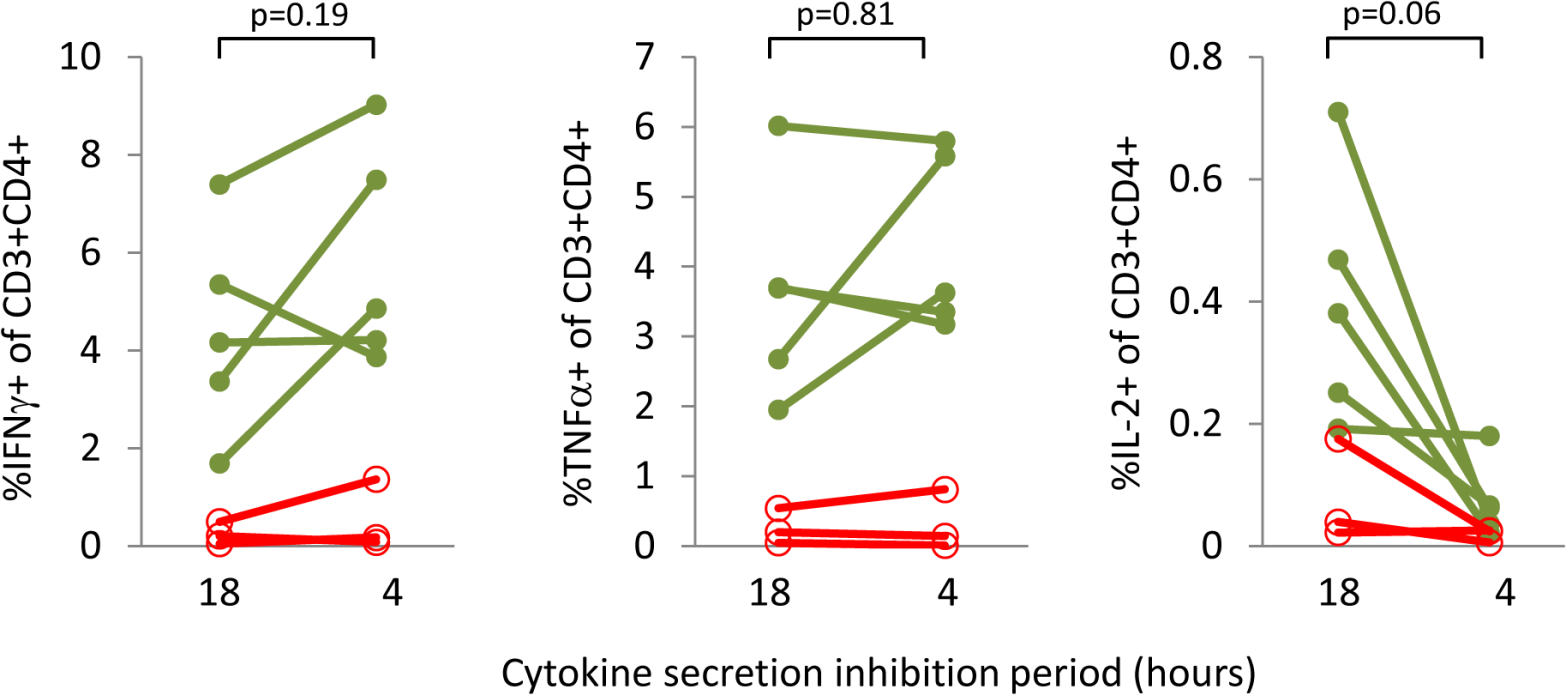
The impact of cytokine secretion inhibition period on the magnitude of intracellular cytokine responses detected in CD4^+^ T-cells. PBMC were stimulated with PPD for 5 days. BFA and monensin were added for the final 18 hours or final 4 hours of the stimulation period. Y-axes represent the percent of live, single CD3^+^CD4^+^ lymphoid cells that are positive for the indicated cytokine. Responses are shown for individuals with latent TB infection (green lines, closed circles; n = 5) or from healthy, uninfected individuals (red lines, open circles; n = 3). Statistical analysis is by Wilcoxon matched pairs signed rank test.

**Fig 7:**
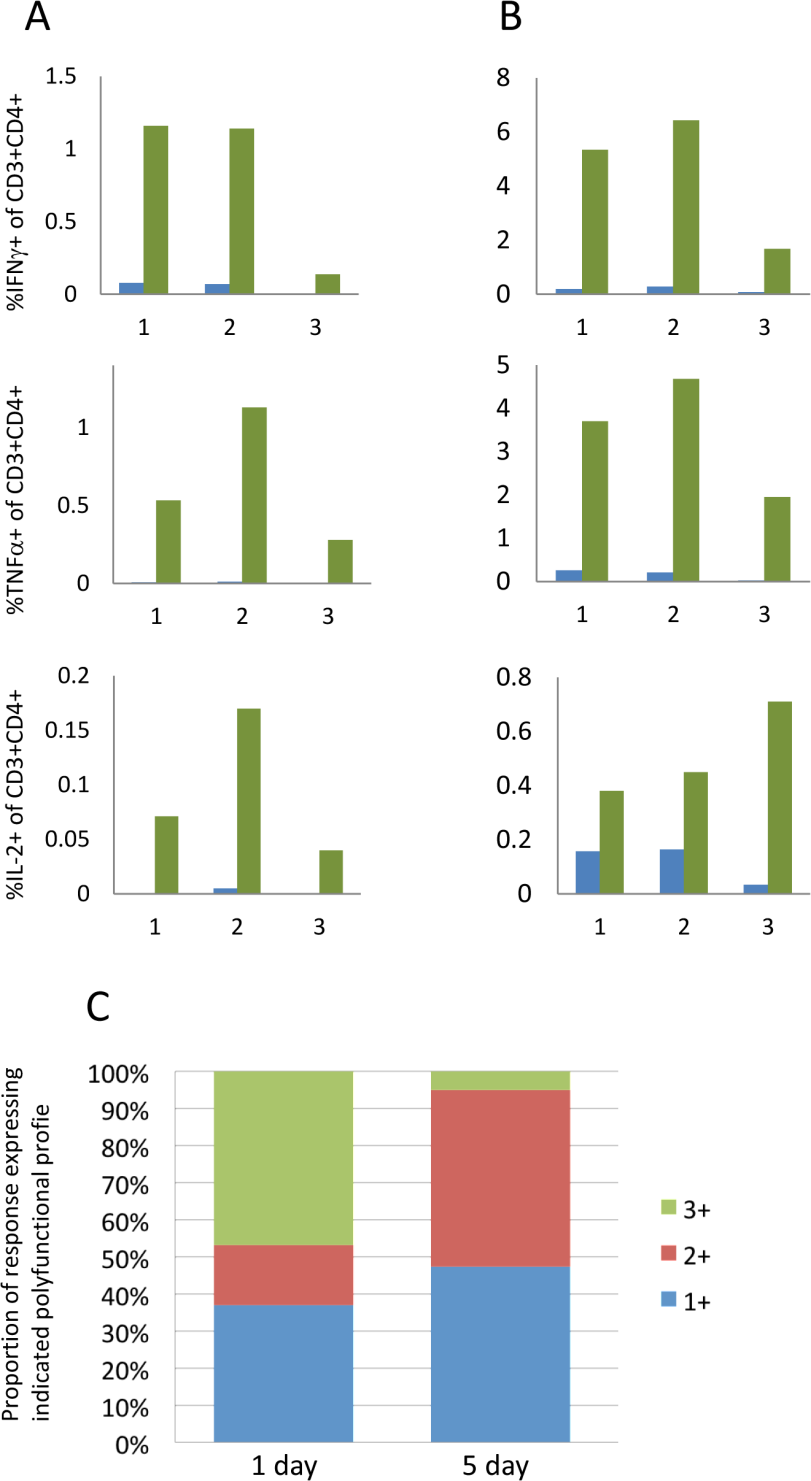
The impact of stimulation period on the magnitude of intracellular cytokine responses detected and polyfunctional profile of CD4+ T-cells. PBMC were stimulated with either HBHA (A) or PPD (B) for 1 day (blue bars) or 5 days (green bars). BFA and monensin were added for the final 18 hours. Results are shown for 3 individuals indicated on the X axes. Y axes represent the percent of live, single CD3^+^CD4^+^ lymphoid cells that are positive for the indicated cytokine. (C) Boolean gating was used to determine the polyfunctional profile of cells responding to PPD following 1 day or 5 days of stimulation. The key refers to the number of cytokines co-expressed by the proportion of cells represented (green, triple positive cells; red, double positive cells; blue, single positive cells).

We have found that for cytokines where the rate of expression is slower, or for which the period of expression is short or intermittent, a longer secretion inhibition time can improve their detection. For example, Fig 6 demonstrates that whereas the cytokines IFNγ and TNFα are detectable with both shorter (4 hour) and longer (18 hour) brefeldin A/monensin incubation periods, IL-2 frequencies show a trend towards a decrease with the shorter inhibition period, although this does not reach statistical significance. Extending the overall stimulation period may also increase the sensitivity of ICS assays. For weaker immunogens or for very low frequency responder cells, a period of 5 days allows for some proliferation of responder T-cells, making them more easily detectable. Another point to consider is that longer assays may allow for the detection of ‘resting’ or central memory T-cell responses that require more time to become activated than 24 hours. This is akin to the so-called cultured ELISpot approach for the detection of memory T-cell responses [33]. In contrast, it has been proposed that polyfunctional T-cells detected in short-term assays are end stage effector cells [34]. Fig 7 shows that for a weaker immunogen (heparin-binding haemagglutinin; HBHA, Fig 7A) and a stronger one (*M*. *tuberculosis* purified protein derivative; PPD, Fig 7B) three cytokines (IFNγ, TNFα and IL-2) are all detectable at greater magnitudes after 5 day stimulations as opposed to 1 day stimulations. The limitations of this approach, however, are illustrated in Fig 7C: triple positive (IFNγ+TNFα+IL-2+) T-cells are more evident after 1 day as compared to 5 day stimulation. Polyfunctional T-cells, detectable early in a stimulation period, appear to reduce or lose completely the expression of one or more cytokines over time.

**Fig 8:**
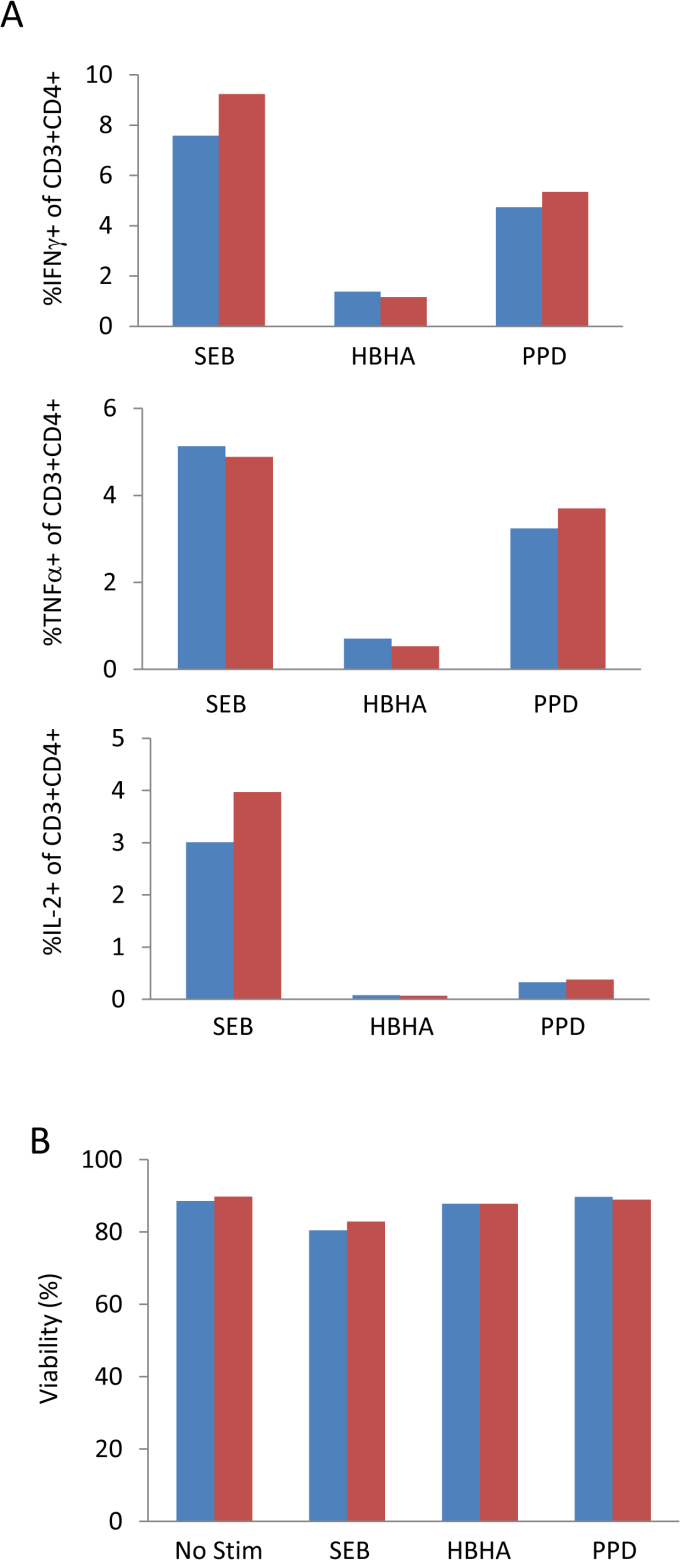
Alternative combinations of cytokine secretion inhibitors have minimal effect on cell viability or the magnitude of responses detected: PBMC were incubated for 5 days with a variety of stimuli. BFA alone (blue bars) or BFA + monensin (red bars) were added for the final 18 hours of the stimulation period. A) The magnitude of cytokine response for each cytokine inhibition condition. B) Cell viability following each cytokine inhibition condition was determined by cell counts prior to and following the inhibition period and percent viability calculated.

Another factor to consider with respect to the inhibition of cytokine secretion is the inhibitor used. The two most commonly used reagents are brefeldin A and monensin. Despite evidence that the secretion of certain cytokines is inhibited better by one or another of these reagents [35]or sometimes a combination of the two together, we found that brefeldin A inhibited secretion as well on its own as in combination with monensin in response to a variety of antigen and control stimuli (Fig 8A). We did not observe any difference between brefeldin A or brefeldin A + monensin in terms of cell viability following the incubation period (Fig 8B). It should be noted that we only considered IFNγ, TNFα and IL-2. We cannot rule out that a combination of secretion inhibitors will perform better for other cytokines of interest and urge investigators to perform comparative analysis for their own cytokines of interest.

In summary, with respect to stimulation and inhibition of cytokine secretion, if the sensitivity of the assay to detect individual cytokines secreted by T-cells is a priority, as in the case of a weaker antigen stimulus for example, a longer assay period such as 5 days with the final 18 hours in the presence of Golgi inhibition is recommended. However if the primary objective is to demonstrate whether or not a vaccine induces a polyfunctional T-cell response consisting of 3 or more cytokines including IL-2, we suggest a shorter, overnight or 1 day assay period with the final 18 hours of this stimulation in the presence of Golgi inhibition. In our hands, this assay approach improved the detectability of these polyfunctional cells.

The nature of the antigen stimulant to be used is also relevant to this discussion, as this will affect the choice of stimulation and Golgi inhibition periods. For example, peptide antigens that bind directly to MHC molecules do not require any processing and so Golgi inhibition can commence at the same time as antigen stimulation whereas a protein antigen or live organism stimulant such as BCG will require a longer period of antigen processing and loading of MHC molecules before a Golgi secretion inhibitor may be added as adding the inhibitor too early would prevent proper antigen processing from taking place [35]. It should be noted that this step should be optimised for each study as very rapidly released immune effector molecules may be missed if the Golgi inhibitor is added too late.

#### Co-stimulatory antibodies

**Fig 9:**
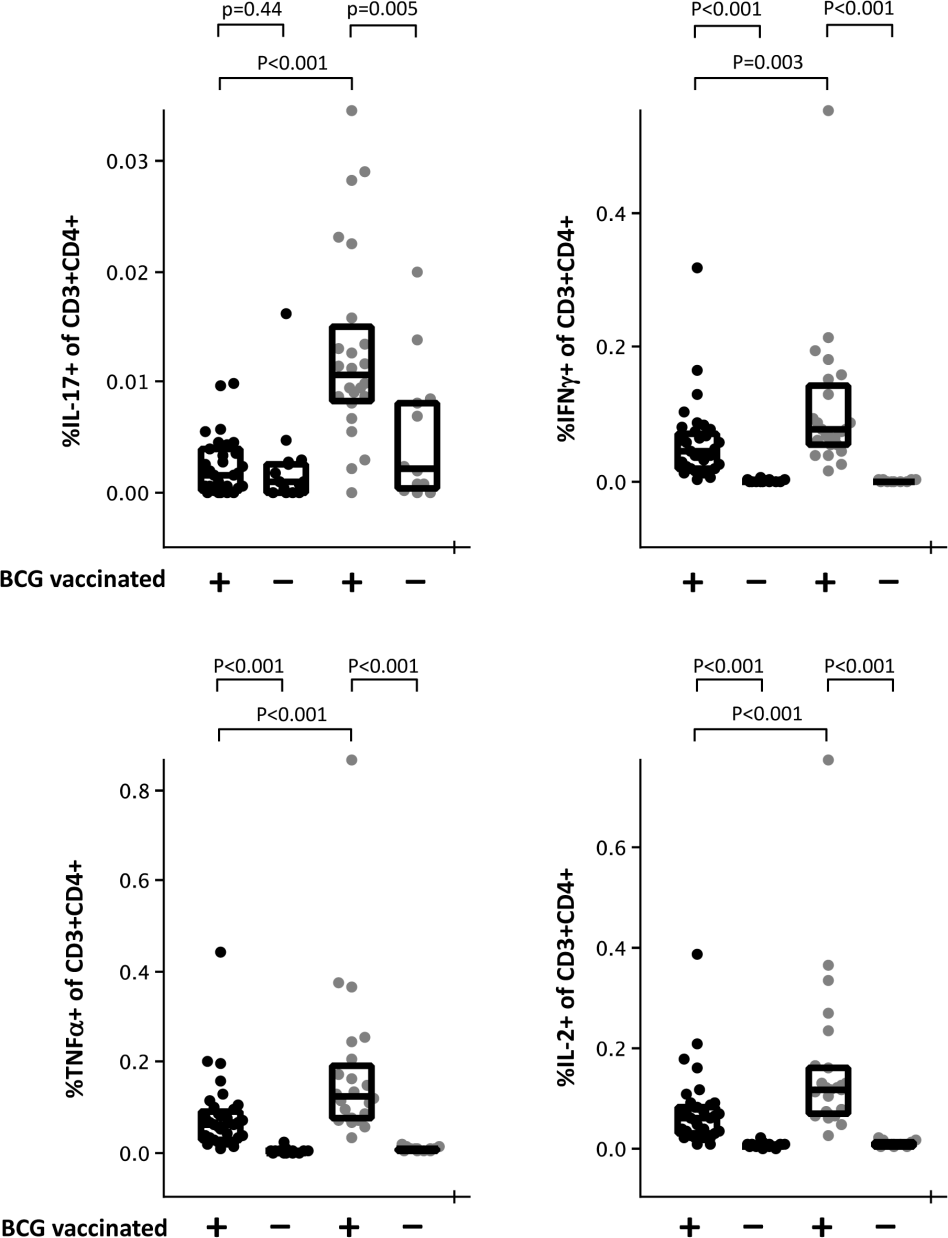
The effect of anti-CD28 and anti-CD49d co-stimulatory antibodies on the detection of antigen-specific cytokine responses. Venous blood samples from BCG vaccinated (+) or unvaccinated (-) infants were diluted 1:1 in Iscove’s modified Dulbecco’s medium and stimulated for 18 hours with PPD in the absence (black dots) or presence (grey dots) of co-stimulatory antibodies. Brefeldin A was added after 2 hours. Y axes represent the percent of live, single CD3^+^CD4^+^ lymphoid cells that are positive for the indicated cytokine. Statistical analysis is by Mann-Whitney U test.

It is well established that the analysis of T-cell immunity *in vitro* can be achieved via the provision of cognate antigen in the form of protein or peptide, i.e. signal 1. In these circumstances, effector T-cells will secrete cytokines, allowing their detection in appropriate assays. However, not all T-cell responses are equal and there can be heterogeneity in intrinsic activation thresholds of different clonal T-cell populations. These differences are manifest in the requirements for more or less of signal 2 in different T-cell responses [36]. This phenomenon has led to the inclusion of co-stimulatory antibodies (against the T-cell B7 receptor CD28 and the integrin α subunit of the VLA-4 receptor CD49d) in ICS assays for the detection of T-cell responses in order to increase the sensitivity of these assays [28]. The experience of the ICS working group suggests that for very low frequency responses, the inclusion of co-stimulatory antibodies can bring the response into a detectable range. For stronger responses however, the effect appears to be to increase the magnitude of the response detectable or in some cases to have no obvious effect. For example, Fig 9 shows that in recently BCG-vaccinated infants stimulation with PPD, anti-CD28 and anti-CD49d significantly increases the magnitude of IL-17 secreting CD4^+^ T-cells that are detected (p<0.001). Only when co-stimulatory antibodies are included is a significant difference in these cells detectable between vaccinated and unvaccinated infants. In contrast, IFNγ^+^, TNFα^+^ and IL-2^+^ CD4^+^ T-cell responses are all detectable in vaccinated but not unvaccinated infants after stimulation with PPD alone albeit at significantly lower frequencies than are seen when co-stimulatory antibodies are included. If sensitivity is of utmost importance we therefore recommend the inclusion of co-stimulatory antibodies. It should be borne in mind however that stimulation with antibodies alone may, in some circumstances, be enough to induce cytokine secretion [37] which might cause an issue of sensitivity in some antigen specific T-cell assays due to increasing background values. For example, we found that compared to unstimulated controls, the addition of co-stimulatory antibodies alone increased the frequency of IL-17^+^ CD4^+^ T-cells in samples from recently BCG-vaccinated infants (0.002% (0.001%-0.004%) and 0.004% (0.003%-0.040%) respectively (p<0.001) (median (lower quartile-upper quartile). This effect was not seen for IFNγ, TNFα or IL-2 responses (data not shown).

#### Post-stimulation fixation and cryopreservation

As mentioned above, it is possible, following sample stimulation and Golgi inhibition, to halt the assay and cryopreserve samples immediately prior to staining and acquisition. This allows for some of the convenience and advantages of using cryopreserved PBMC as the starting material (i.e. batched processing; transport of samples and use of a centralized facility for staining and acquisition; more user-friendly timing of assays) but with the additional flexibility to use fresh whole blood for stimulations and a number of studies have used this approach successfully [28,30–32]. Usually, following stimulation, a reagent that combines red blood cell lysis with formaldehyde-mediated sample fixation is used after which the remaining white cells are pelleted and frozen directly in cryo-vials. As epitopes on cells following fixation of stimulated whole blood may have changed, antibody clones should be selected and tested carefully. If fixation is used, an amine-reactive live/dead stain cannot be used as part of the panel as these reagents do not work on fixed cells. If a live/dead stain was required, red cell lysis may be carried out using an ammonium chloride based solution and the stained performed prior to fixation and cryopreservation.

#### Live/dead cell discrimination

Often, the population of interest in immuno-monitoring assays such as ICS/flow cytometry represents a very small proportion of the total cell population. It is important therefore to maximize the sensitivity of the assay in order to allow the discrimination of positive events. A careful acquisition and gating strategy is essential towards this end and some of the techniques available that utilize acquisition and analysis software settings to “clean up” the signal will be addressed below. Cell staining however provides an early opportunity to minimize assay background in the form of a stain for dead or dying cells that may later be excluded from the analysis [38]. It is also possible to include in the staining panel, antibodies against markers of cells that are not to be analysed but that may non-specifically bind cytokine-specific antibodies leading to background noise [38]. CD19 and CD14 have been used in this regard to exclude B-cells and monocytes respectively from T-cell analysis panels. If these antibodies are conjugated to a fluorophore that emits in the same channel as the dead cell stain, a “dump” channel is created for the combined exclusion of events that could contribute to reduce noise in the assay. The main drawback to the inclusion of a dump channel is the use of a detector that may otherwise have been used as an extra analysis channel which, in situations where flow cytometers with fewer detectors are available, might be prohibitively limiting. Also, as mentioned above, amine-reactive dye-based viability stains cannot be used on fixed cells. However, for the discrimination of rare events, the exclusion of potential sources of background noise is a powerful technique and we would recommend the use of a viability stain in most circumstances as part of a dump channel, as beneficial to the quality, and reproducibility of the work/analysis. Of course, it will be necessary to fit such a dump channel into the overall antibody staining panel used in a given study. Although an in-depth discussion of the decisions relating to antibody choice are beyond the scope of this article, we refer the reader to the excellent and comprehensive review by Mahnke and Roederer on this topic [39].

#### Sample acquisition, gating strategies and analysis

Reliable gating strategies and analysis of ICS experiments are underpinned by high quality raw data. This is achieved in part by optimizing protocols to obtain the highest signal to background ratio, as described in this article. To this may be added optimization of instrument set-up to ensure standardized performance [40], the need for careful compensation between fluorescence channels, the use of fluorescence-minus-one (FMO) control stains and for the acquisition of an adequate number of positive events. The latter point is a particular challenge when sample material or the number of detection channels available on a flow cytometer is limiting. The desire to extract the maximum amount of information from a blood sample may lead to its splitting into multiple stimulation tubes which may be further split for staining with different antibody panels. Each of these decisions ultimately results in fewer cells available for final acquisition. However, if the frequency of the cell population of interest is very low, as is often the case in ICS experiments, it is vital for the production of statistically meaningful data that a sufficient number of positive events are collected. There is no universal rule as to what this number is. It can only be determined for each experiment with a knowledge of the expected distribution of frequencies of the population of interest in an appropriate set of samples from a negative control (e.g. unvaccinated) cohort [41].

In addition to steps such as a dead cell exclusion stain, it is possible to use flow cytometry analysis software to exclude further events that may be a source of noise. For example, cells that stick to each other and are acquired as a doublet can distort data but may be removed from the analysis by the use of a gate defined by the FSC-A and FSC-H parameters that includes only single cells. Other anomalies in the acquisition signal, due to bubbles for example, may be visualized when events are displayed as a function of the time parameter and these can again be excluded by careful gating. Finally, most ICS experiment analysis involves the positioning of phenotypic gates to isolate cell subsets of interest such as CD3^+^CD4^+^ events (helper T-cells) or CD3^+^CD8^+^ events (cytotoxic T-cells), prior to the final addition of a cytokine gate to derive the frequency of cytokine-positive events. Although most investigators would agree on the phenotypic markers that are required for the identification of T-cell subsets, the exact positioning and hierarchy of placement of the gates that define these markers differs from person to person based on lab convention, prior training or personal preference. Although the variations in data output that these different approaches produce are not prohibitive, they are avoidable, providing a common gating strategy is agreed upon and used from the outset. To this end, previous, large scale efforts to identify effective gating approaches should be heeded [42] as should additional guidelines that govern the appropriate reporting of these methodological and analytical details in order to improve clarity and promote a consensus approach in the field; for example The Minimal Information about T-Cell Assays (MIATA) project [5].

## Conclusions

As with protocols and standard operating procedures for other assays of T-cell function, those for ICS/flow cytometry have often arisen in individual, specialist laboratories and been designed to work best for a specific application. For example, incubation periods might have been optimized for stimulation with peptides or a particular culture medium may be used because certain cell populations survive well in that medium, or sometimes just because that is the way a particular laboratory has always done it. We have found that, if different groups across numerous sites and over a period of time are performing ICS assays with the hope of achieving consistency in results, there are certain protocol steps for which different approaches are possible and hence should be agreed in advance. In this paper, we describe several of these steps and make some recommendations where appropriate. The intention is that where groups are performing immune-monitoring of responses to novel vaccines as a part of clinical trials, consistency can be achieved both within a trial and ultimately, even between different trials of separate vaccine candidates. It should be noted that this paper concentrates largely upon CD4+ T-cell responses however it will often be desirable to measure CD8+ T-cells either independently or at the same time as CD4+ T-cells depending upon the vaccine candidate in question and it should not be assumed that assay conditions that suit the detection of the former will always be optimal for detection of the latter. Below are some final ‘open-door’ recommendations for additional points that should be considered in addition to those discussed above.

- Synchronization of sample processing so that all samples are subject to the same time delay between drawing and processing or processing and flow cytometric acquisition
- Consistent analysis throughout a study where possible, ideally by the same person
- Use of one reagent and antibody batch throughout a study and at different sites
- Use of internal controls for validation and maintenance of consistency in assay performance across sites and over time

## Supporting Information

S1 Fig Gating strategy for flow cytometry. Successive gates were applied to exclude dead cells, CD14+ or CD19+ cells, to identify singlet cells, lymphocytes, CD3+ cells and subsequently CD4+ or CD8+ cells. Detailed analysis for CD3+CD4+ and CD3+ CD8+ cells is shown in the middle and lower panels respectively. Frequency of lymphoblasts (left) is defined by the gate based on FSC-SSC. Evaluation of cytokine production, more specifically IFNγ, TNFα and IL-2 following stimulation with PPD, is shown in the right panels and frequencies of cells that produce all possible combinations are calculated using Boolean gates. Fig refer to frequency of corresponding gate or quadrant.
(TIF)

S2 Fig Representative ICS plots for flow cytometry data. Following gating of cells as illustrated in S1 Fig. Frequencies of CD3+CD4+ cells producing different combinations of cytokines were obtained for unstimulated cells in 1 day (A) and 5 day (C) ICS assays and for PPD stimulated cells in 1 day (B) and 5 day (D) ICS assays. Figs refer to the frequency of corresponding quadrant.
(TIF)
