# Supplementary figures and images for: Intracellular Cytokine Staining and Flow Cytometry: Considerations for Application in Clinical Trials of Novel Tuberculosis Vaccines
Source: PLoS One. 2015 Sep 14;10(9):e0138042. doi: 10.1371/journal.pone.0138042 (PMC4569436; doi:10.1371/journal.pone.0138042)

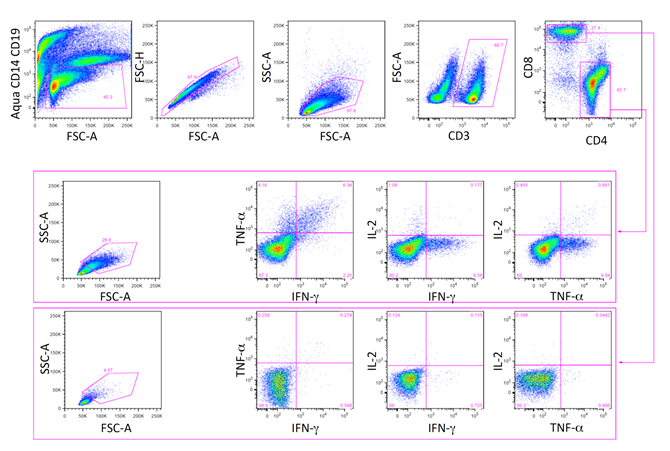

Supplement: S1 Fig — Successive gates were applied to exclude dead cells, CD14+ or CD19+ cells, to identify singlet cells, lymphocytes, CD3+ cells and subsequently CD4+ or CD8+ cells. Detailed analysis for CD3+CD4+ and CD3+ CD8+ cells is shown in the middle and lower panels respectively. Frequency of lymphoblasts (left) is defined by the gate based on FSC-SSC. Evaluation of cytokine production, more specifically IFNγ, TNFα and IL-2 following stimulation with PPD, is shown in the right panels and frequencies of cells that produce all possible combinations are calculated using Boolean gates. Fig refer to frequency of corresponding gate or quadrant. (TIF) [file pone.0138042.s001.tif]

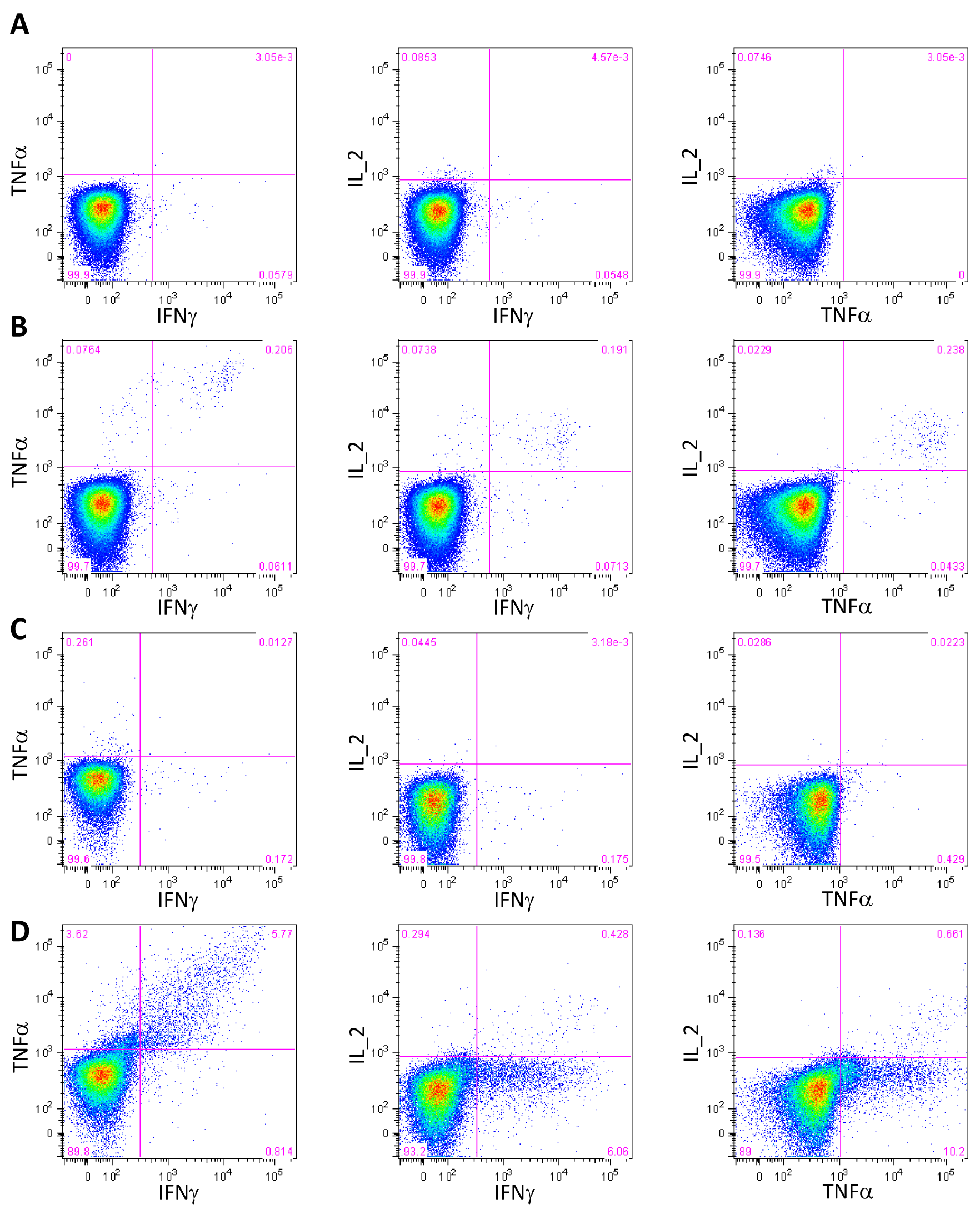

Supplement: S2 Fig — Following gating of cells as illustrated in S1 Fig. Frequencies of CD3+CD4+ cells producing different combinations of cytokines were obtained for unstimulated cells in 1 day (A) and 5 day (C) ICS assays and for PPD stimulated cells in 1 day (B) and 5 day (D) ICS assays. Figs refer to the frequency of corresponding quadrant. (TIF) [file pone.0138042.s002.tif]
